# Nodularin‐R Synergistically Enhances Abiraterone Against Castrate‐ Resistant Prostate Cancer via PPP1CA Inhibition

**DOI:** 10.1111/jcmm.70210

**Published:** 2024-11-17

**Authors:** Yiqiao Huang, Yi Cen, Hualing Wu, Guohao Zeng, Zhengming Su, Zhiming Zhang, Shourui Feng, Xianhan Jiang, Anyang Wei

**Affiliations:** ^1^ Department of Urology, Nanfang Hospital Southern Medical University Guangzhou China; ^2^ Department of Urology, Key Laboratory of Biological Targeting Diagnosis, Therapy and Rehabilitation of Guangdong Higher Education Institutes The Fifth Affiliated Hospital of Guangzhou Medical University Guangzhou China; ^3^ Guangdong Provincial Key Laboratory of Molecular Target & Clinical Pharmacology, the NMPA and State Key Laboratory of Respiratory Disease Guangzhou Medical University Guangzhou China; ^4^ Department of Gynecology, Key Laboratory of Biological Targeting Diagnosis, Therapy and Rehabilitation of Guangdong Higher Education Institutes The Fifth Affiliated Hospital of Guangzhou Medical University Guangzhou China; ^5^ School of Life Sciences Sun Yat‐sen University Guangzhou China

**Keywords:** abiraterone, castration‐resistant prostate cancer, CRISPR/Cas9, nodularin‐R, PPP1CA

## Abstract

Clinically, most prostate cancer (PCa) patients inevitably progress to castration‐resistant prostate cancer (CRPC) with poor prognosis after androgen deprivation therapy (ADT), including abiraterone, the drug of choice for ADT. Therefore, it is necessary to explore the resistance mechanism of abiraterone in depth. Genome‐wide CRISPR/Cas9 knockout technology was used to screen CRPC cell line 22Rv1 for abiraterone‐resistant genes. Combined with bioinformatics, a key gene with high expression and poor prognosis in CRPC patients was screened. Then, the effects of target gene on abiraterone‐resistant 22Rv1 cell function were explored by silencing and overexpression. Further, a natural product with potential targeting effect was identified and validated by molecular docking and protein expression. Molecular dynamics simulations revealed potential mechanism for the natural product affecting target protein expression. Finally, the combined anti‐CRPC effects of the natural product and abiraterone were validated by cellular and in vivo experiments. Five common resistance genes (KCNJ3, COL2A1, PPP1CA, MDH2 and EXOSC5) were identified successfully, among which high PPP1CA expression had the worst prognosis for disease‐free survival. Moreover, PPP1CA was highly expressed in abiraterone‐resistant 22Rv1 cells. Silencing PPP1CA increased cell sensitivity to abiraterone while promoting apoptosis and inhibiting clone formation. Overexpressing PPP1CA exerted the opposite effects. Molecular docking revealed the binding mode of the natural product nodularin‐R to PPP1CA with a dose‐dependent manner for inhibition. Mechanistically, nodularin‐R attenuates the interaction between PPP1CA and USP11 (deubiquitinating enzyme), potentially promoting PPP1CA degradation. Additionally, combination of 2.72 μM nodularin‐R and 54.5 μM abiraterone synergistically inhibited the resistant 22Rv1 cell function. In vivo experiments also revealed that combination therapy significantly inhibited tumour growth and reduced inducible expression of PPP1CA. PPP1CA is a key driver for abiraterone resistance, and nodularin‐R enhances the anti‐CRPC effects of abiraterone by inhibiting PPP1CA.

**Abbreviations**
ACTB: actin beta
ADT: androgen deprivation therapy
AR: androgen receptor
BP: biological process
CC: cellular component
CI: combination index
COL2A1
: collagen type II alpha 1 chain
CRISPR: clustered regularly interspaced short palindromic repeats
CRPC: castration‐resistant prostate cancer
CRSC: castration‐sensitive prostate cancer
DEGs: differentially expressed genes
DFS: disease‐free survival
EXOSC5
: exosome component 5
FC: fold change
GEO: Gene Expression Omnibus
GEPIA: gene expression profiling interactive analysis
GO: gene ontology
HE: haematoxylin–eosin
hGeCKO: human CRISPR knockout library
HR: hazard ratio
IC_50_
: half maximal inhibitory concentration
IHC: immunohistochemical
KCNJ3
: potassium inwardly rectifying channel subfamily J member 3
KEGG: Kyoto Encyclopedia of Genes and Genomes
MC: molecular function
MDH2
: malate dehydrogenase 2
MM/PBSA: molecular mechanics/Poisson‐Boltzmann surface area
OD: optical density
PCa: prostate cancer
PDB: Protein Data Bank
PP: protein phosphatase
PPI: protein–protein interactions
PPP1CA
: protein phosphatase 1 catalytic subunit alpha
PRAD: prostate adenocarcinoma
qRT‐PCR: quantitative reverse transcriptase PCR
RI: resistance index
RMSD: root‐mean‐square deviation
RMSF: root‐mean‐square fluctuation
RRA: Robust Rank Aggregation
SASA: solvent‐accessible surface area
sgRNAs: single‐guide RNAs
siRNAs: small interfering RNAs
STP: Swiss Target Prediction
USP11: ubiquitin specific peptidase 11
WB: western blot

## 1 Introduction

Prostate cancer (PCa) is one of the most prevalent malignant tumours in men, and its morbidity and mortality are related to the age of patients, with the highest incidence in patients over 65 years old [1]. Most PCa patients have metastases at the initial diagnosis, for which androgen deprivation therapy (ADT) is preferred [2]. Studies have found that 80% of PCa patients respond positively to treatment but inevitably progress to castration‐resistant prostate cancer (CRPC), with 1/3 of these patients becoming resistant within 1–2 years [3]. Until 2010, docetaxel was the only drug prolonging survival in CRPC patients [4]. With the increasing awareness of CRPC, some novel ADT drugs, such as abiraterone, are starting to be applied.

Abiraterone is a novel endocrine drug for CRPC treatment, which can selectively inhibit CYP17 enzyme and block androgen synthesis in adrenal, testicular and prostate tissues, and then suppress tumour growth [5]. Due to the large individualised differences in CRPC, the drug efficacy will vary among individuals, which is related to abiraterone resistance [6, 7]. Abiraterone‐resistant mechanisms are complex including androgen receptor (AR) splice variant production, increased AR copy number, enhanced AR post‐translational modification, AR mutation, AR deletion and AR‐independent signalling pathway activation [8, 9, 10, 11, 12]. Altered AR status leading to resistance as abiraterone loses its target is an inevitable disease process. Therefore, it is vital to find other key regulators driving CRPC to ameliorate abiraterone resistance.

Nowadays, high‐throughput genome‐wide screening and sequencing provide potent technical support for revealing the drug‐resistant mechanisms of various diseases, in which the CRISPR/Cas9 system is widely used as an efficient gene editing tool [13, 14, 15, 16]. The aim of CRISPR/Cas9 screening in oncology is to identify genotype‐specific weaknesses, and targeted deletion of these genes can reduce the viability of cancer cells, thus providing potential therapeutic targets. Additionally, combining CRISPR/Cas9 screening with drugs can better understand how tumours respond to drug therapy.

To comprehensively understand the resistance characteristics in PCa, we selected CRPC cell line 22Rv1 for genome‐wide CRISPR/Cas9 screening, and collected the surviving cells for high‐throughput sequencing after three rounds with abiraterone treatment. A key resistance gene was further mined in combination with bioinformatics. Then, a natural small molecule product with inhibitory effect was identified based on the properties of target protein. The synergistic inhibitory effects of the natural products with abiraterone were further observed in resistant 22Rv1 cells. Finally, the combined therapeutic effects were validated in vivo.

## 2 Materials and Methods

### 2.1 Cell Line Culture

Sensitive 22Rv1 cell line was provided by Boyao Biotechnology (Guangzhou, China). Cells were cultured in Dulbecco's modified Eagle medium (DMEM) (Gibco, USA) containing 10% foetal bovine serum (FBS) (Gibco, USA) and 100 mg/L penicillin/streptomycin (Gibco, USA) in a humidified condition at 37°C with 5% CO_2_.

### 2.2 Genome‐Wide CRISPR Screening and sgRNA Deep Sequencing

The human CRISPR knockout library (hGeCKO) (Addgene, #1000000048) was used to identify genes responsible for abiraterone resistance in 22Rv1 cells. The hGeCKO library plasmid contains 123,411 different single‐guide RNAs (sgRNAs) targeting 19,050 genes (6 sgRNAs per gene) and 1864 miRNAs (4 sgRNAs per miRNA) at a low MOI (~0.5) to ensure most cells received only one stably integrated RNA guide. hGeCKO library‐edited 22Rv1 cells were constructed as previously described by Dr. Feng Zhang [17]. Transduced cells were screened with puromycin (MCE, USA) for 15 days to generate a mutant cell pool. The optimal screening concentration (50% viability inhibition) for abiraterone (MCE, USA) was identified as 20 μM by cell activity assay (Figure S1). After three consecutive rounds of cell treatment with 20 μM abiraterone, surviving cells were collected and genomic DNA was isolated using the HiPure Tissue DNA Mini Kit (Magen, China). Inserted sgRNA sequences were amplified and sequenced using next‐generation sequencing on an Illumina HiSeq2500 system by Novogene Technology (Beijing, China). Genome‐wide CRISPR screening coverage was shown in Table S1. Differentially expressed genes (DEGs) with |log2 (fold change, FC)| > 0 and *p* < 0.05 were screened out. MAGeCK was employed to analyse the CRISPR screen results, where sgRNAs targeting specific genes were ranked based on the Robust Rank Aggregation (RRA) score, with smaller RRA scores indicating higher significance. The top resistance genes were identified by evaluating both the total read counts of sgRNAs and the diversity of sgRNAs (i.e., the number of distinct sgRNAs detected that target the same gene).

### 2.3 KEGG And GO Enrichment Analysis

R package (clusterProfiler) was applied to perform Kyoto Encyclopedia of Genes and Genomes (KEGG) pathway and Gene Ontology (GO) functional enrichment analysis for DEGs respectively. KEGG exhibited the top 15 significant pathways. GO consisted of biological process (BP), cellular component (CC) and molecular function (MC), each exhibiting the top 5 terms. All up‐regulated DEGs enriched in KEGG and GO were considered as candidate genes. *p* < 0.05 was considered statistically significant.

### 2.4 Key Resistance Gene Screening

RNA expression profiles were obtained from the GSE221961 dataset in the Gene Expression Omnibus (GEO) database (https://www.ncbi.nlm.nih.gov/geo/). The GSE221961 dataset contained transcript expression data from tissues of three castration‐sensitive prostate cancer (CRSC) and CRPC patients each. The available sample data were analysed using the GEO2R platform. DEGs with |log2 (FC)| > 1 and *p* < 0.05 were screened out according to the Benjamini–Hochberg method. Key resistance genes were screened by interacting up‐regulated DEGs between CRISPR screening and GSE221961 dataset using VENNY 2.1 tool (https://bioinfogp.cnb.csic.es/tools/venny/index.html). Related volcano diagram and heat map were plotted using R package (ggplot2).

### 2.5 Gene Prognosis and Expression Analysis

The Gene Expression Profiling Interactive Analysis (GEPIA) database (http://gepia.cancer‐pku.cn/) was utilised to analyse the effect of key resistance gene expression on disease‐free survival (DFS) in prostate adenocarcinoma (PRAD) patients (log‐rank test), as well as their expression levels in PRAD patients (tumour, *n* = 492; normal, *n* = 192). Hazard ratio (HR) > 1 was considered a risk factor. *p* < 0.05 was considered statistically significant.

### 2.6 Cell Viability Assay

Cells were seeded into 96‐well culture plate at 5 × 10^3^ cells/well, and 100 μL serum medium was added and cultured for 12 h. After culturing for 48 h, 10 μL CCK‐8 reagent (Beyotime, China) was added, and the optical density (OD) of the cells was detected at 450 nm. The cell viability at each drug concentration was calculated according to the OD. The combined anti‐tumour effect of abiraterone and nodularin‐R was evaluated using the combination index (CI) proposed by Chou‐Talalay [18]. The cell growth curve was plotted and the half maximal inhibitory concentration (IC_50_) was calculated using the viability as the vertical coordinate and lg (concentration) as the horizontal coordinate.

### 2.7 Resistant 22Rv1 Cell Line Construction

Briefly, drug‐sensitive 22Rv1 cells were used as the parental cells, and their IC_50_ was determined (IC_50_ = 16.13 μM). During the logarithmic growth phase, abiraterone was added at a starting concentration that was 1/5 of the IC_50_ value of the parental cells. Once the cell density reached 50%, the medium was replaced with drug‐free medium. When the cell density recovered to 80%, the previous drug treatment cycle was repeated for 6–8 rounds over a period of 6 months. After 6 months, we obtained a low‐resistance cell line (IC_50_ = 42.2 μM). Moderate resistance cell lines (IC_50_ = 117 μM) were developed once the cells could grow stably under higher abiraterone concentrations, with a final concentration equivalent to two‐fold the IC_50_ of the parental cells, for an additional 6 months. Under these conditions, both cell lines exhibited morphological changes, including a loss of tight cell‐to‐cell contacts, resulting in a more scattered growth pattern. Additionally, the cells temporarily developed arm‐like projections, indicating changes in their structural organisation. The drug‐resistant cell lines were detected, and the resistance index (RI) was calculated. RI = IC_50_ of drug‐resistant cell line/IC_50_ of parental cell line (1 < RI ≤ 5 for low resistance, 5 < RI ≤ 15 for moderate resistance and RI > 15 for high resistance).

### 2.8 Small Interfering RNA (siRNA) Construction and Transfection

siRNA specific to PPP1CA for silencing its expression (siPPP1CA), including non‐targeting control (siNC), were synthesised by GENERAL BIOL (Anhui, China). Cells were seeded in 6‐well culture plate at 1 × 10^6^ cells/mL and transiently transfected with 2 mL OPTI‐MEM medium (Gibco, USA) using siRNA transfection reagent (Santa Cruz, USA). siPPP1CA sequences: 5′‐GCUCUUUCCAGAUCCUCAATT‐3′ (F) and 5′‐UUGAGGAUCUGGAAAGAGCTT‐3′ (R). siNC sequences: 5′‐GUAUGACA ACAGCCUCAAGTT‐3′ (F) and 5′‐CUUGAGGCUGUUGUCAUACTT‐3′ (R).

### 2.9 Plasmid Construction and Transfection

pIRES2‐ZsGreen1‐PPP1CA plasmid was synthesised by GENERAL BIOL (Anhui, China) for overexpressing PPP1CA (oePPP1CA) with empty plasmid as control (oeNC). Cells were seeded in 6‐well culture plate 1 × 10^6^ cells/mL with 2 mL serum medium. Lipofectamine 2000 (Thermo Fisher, USA) was used for transfection according to manufacturer's instructions.

### 2.10 Cell Apoptosis Assay

The procedure was performed according to the operating instructions of the Annexin V‐FITC/PI apoptosis kit (Beyotime, China). After transfection for 48 h, cells were collected at 1 × l0^6^ cells/tube, and 5 μL Annexin V‐FITC and 5 μL PI reagents were added sequentially and incubated for 15 min protected from light. The cell fluorescence was detected at 488 nm of excitation light (red fluorescence for PI and green fluorescence for Annexin V‐FITC). The apoptotic cell number was counted and the apoptosis rate was calculated.

### 2.11 Cell Clone Formation Assay

Cells were seeded in 6‐well plate at 200 cells/well with 2 mL serum medium. The plate was gently shaken to disperse the cells and incubated in incubator for about 2 weeks. The culture was terminated when visible clonal spots appeared, fixed with 4% paraformaldehyde (Beyotime, China) and stained with 0.5% crystal violet (BOSTER, China). The clone formation rate was calculated by counting the number of clonal spots under the microscope.

### 2.12 Molecular Docking and Molecular Dynamics Simulation

Docking simulations between nodularin‐R and PPP1CA were conducted using Autodock Vina (v1.5.7). The initial model of PPP1CA was obtained from the Protein Data Bank (PDB) database (https://www.rcsb.org/). The structure of nodularin‐R was retrieved from the PubChem database (https://pubchem.ncbi.nlm.nih.gov/), and its 3D structure was generated and energy‐minimised using Chem3D software (v20.0.0.41). Structural figures were prepared in PyMoL, UCSF Chimera and UCSF ChimeraX. 2D ligand‐protein interaction diagram was generated using LigPlot^+^ software (v2.2).

Molecular dynamics simulations were conducted using Gromacs2022.3 software to investigate the impact of nodularin‐R on the interaction between USP11 and PPP1CA [19]. The initial model of USP11 was obtained from the PDB database and PPP1CA model was obtained from molecular docking result. The protein dockings was conducted with GRAMM Web Server database (https://gramm.compbio.ku.edu/). Prior to simulation, small molecules were preprocessed using AmberTools22, where GAFF force field was added, and hydrogenation and RESP potential calculation were performed using Gaussian 16 W. The simulations were carried out under a static temperature of 300 K and atmospheric pressure (1 Bar). The Amber99sb‐ildn force field was employed for the protein, while water molecules were utilised as the solvent with the Tip3p water model. The total charge of the simulation system was neutralised by adding an appropriate number of Na^+^ ions. The energy minimisation of the system was performed using the steepest descent method. Subsequently, equilibrium simulations were conducted under the isothermal isovolumic ensemble and isothermal isobaric ensemble for 100,000 steps each, with a coupling constant of 0.1 ps and a duration of 100 ps. Following equilibrium, free molecular dynamics simulations were carried out for a total of 5,000,000 steps with a step length of 2 fs, resulting in a total duration of 100 ns. After completion of the simulations, trajectory analysis was performed using built‐in tools within the software. Key analyses included root‐mean‐square deviation (RMSD), root‐mean‐square fluctuation (RMSF), solvent‐accessible surface area (SASA) and protein rotation radius of each amino acid trajectory. Free energy calculations, molecular mechanics/Poisson‐Boltzmann surface area (MM/PBSA) was performed to indicate free energy binding.

### 2.13 Quantitative Reverse Transcriptase PCR (qRT‐PCR) Assay

Total RNA was extracted from cells using Trizol reagent (BioTeke, China). After determining the concentration and purity of RNA samples, RNA reverse transcription was performed according to the steps of reverse transcription kit (Vazyme, China). The synthetic cDNAs were used as template for fluorescence detection using SYBR Green qPCR detection kit (Biosharp, China). The reaction procedure was as follows: pre‐denaturation at 95°C for 5 min, followed by 40 cycles of denaturation at 95°C for 30 s, annealing at 60°C for 30 s and extension at 72°C for 15 s. The results were quantified using the relative quantitative 2^−∆∆CT^ method. Primer sequences for PPP1CA: 5′‐GTTCCTCCACAAGCACGACT‐3′ (F) and 5′‐AGCATTGTCAAACTC GCCACA‐3′ (R). Primer sequences for ACTB: 5′‐TGGCACCCAGCACAATGAA‐3′ (F) and 5′‐CTAAGTCATAGTCCGCCTAGAAGCA‐3′ (R).

### 2.14 Western Blot (WB) Assay

Cells or tissues were lysed in RIPA lysis buffer (Beyotime, China) supplemented with 0.1% protease‐inhibitor (Bioss, China). The 40 μg isolated proteins were separated on 12% SDS gels and electrotransferred to polyvinylidene difluoride membranes. Then, incubation for 14 h with primary antibodies against PPP1CA (1:1500, Abcam, USA) and ACTB (1:1000, Bioss, China). The membrane was washed and incubated in horseradish peroxidase‐conjugated secondary antibody. Antibody‐bond protein bands were assayed using a chemiluminescent luminol enhancer solution (BOSTER, China). The gel imaging system was used for imaging analysis.

### 2.15 Animal Modelling and Therapy

Animal experiments followed the guidelines of the Institutional Animal Care and Use Committee of the Animal Experiment Center of Guangzhou Medical University and the Regulations on the Management of Laboratory Animals Affairs.

Sisteen BALB/c nude male mice (4 weeks age, 20 ± 2 g) were pre‐housed for 7 days at room temperature of 24°C ± 2°C and humidity of 40%–60%. The nude mice were randomly divided into 4 groups (control, abiraterone, nodularin‐R and combination groups, 4 mice each) and continued feeding under the same conditions after ear tagging. The resistant 22Rv1 cell suspension was prepared and inoculated subcutaneously in the right upper limb of nude mice at an inoculum volume of 5 × 10^6^ cells/each (total volume of 200 μL) to construct a subcutaneous tumour model. Modelling was considered successful when the tumour volume was greater than 100 mm^3^, and therapy was initiated at this time. Abiraterone was administered daily by gavage (150 mg/kg/day) in the abiraterone group, nodularin‐R was administered daily by intratumour injection (7.5 mg/kg/day) in the nodularin‐R group, and the combination group was administered concurrently. During the therapy period, the long (*L*) and wide (*W*) diameters of the tumours were measured and the tumour volume (*V* = 0.5 × *L* × *W* ^2^) was calculated every 2 days, and the nude mice were weighed simultaneously. Tumour volume of 2000 mm^3^ was taken as the experimental endpoint, and tumour tissues were taken from nude mice after anaesthetic death, part of which was frozen at −70°C for protein assay, and part of which was fixed with 4% paraformaldehyde (Beyotime, China) for pathological assay.

### 2.16 Haematoxylin–Eosin (HE) Staining

Staining was performed according to the instructions of HE staining kit (Beyotime, China). Steps in brief: the tissue was dehydrated and then embedded in paraffin; paraffin slices (thickness of 5 μm) were deparaffinised with xylene; stained with haematoxylin staining solution for 10 min after ethanol washing; stained with eosin staining solution for 2 min after ethanol washing; slices were sealed after dehydration and transparency; observed and photographed under the microscope.

### 2.17 Immunohistochemical (IHC) Staining

Staining was performed according to the instructions of IHC staining kit (Beyotime, China). Steps in brief: slices were deparaffinised with xylene and rehydrated with ethanol; antigen was repaired with 0.01 mol/L citrate buffer; slices were incubated with 3% hydrogen peroxide for 5 min to eliminate endogenous peroxidase; slices were blocked by adding 50 μL non‐immune animal serum for 30 min; washed in PBS and then incubated with PPP1CA primary antibody (1:200, Abcam, USA) overnight at 4°C; slices were equilibrated at room temperature for 30 min and then incubated with the secondary antibody at 37°C for 30 min; washed in PBS buffer and then subjected to DAB chromatography and haematoxylin staining; slices were dried and sealed with neutral gum; observed and photographed under the microscope.

### 2.18 Statistical Analysis

Statistical analysis was performed by SPSS (v18.0) software. Experimental data were presented as mean ± standard deviation. Comparison between multiple groups was made using one‐way ANOVA (Tukey's posthoc test). *p* < 0.05 was considered statistically significant. Data for cell experiments was collected from three independent replicates, while animal experiments were conducted with four independent replicates.

## 3 Results

### 3.1 Genome‐Wide CRISPR Screening for Abiraterone‐Resistant Genes

**FIGURE 1:**
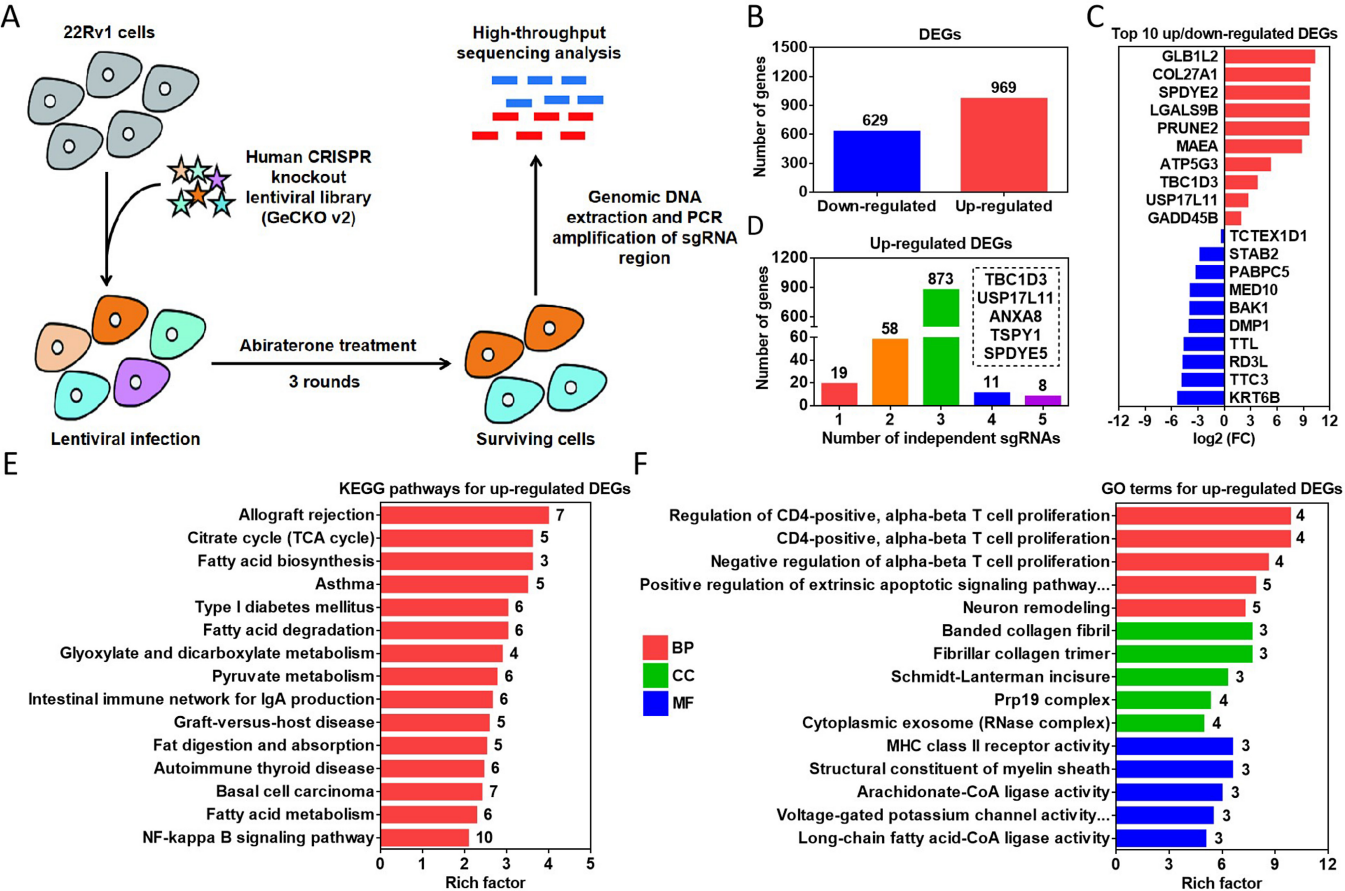
Genome‐wide CRISPR/Cas9 screening for abiraterone‐resistant genes in 22Rv1 cells. (A) Schematic of abiraterone‐resistant 22Rv1 cell construction for high‐throughput sequencing analysis. (B) Number of up‐ (*n* = 969) and down‐ (*n* = 629) regulated DEGs in surviving cells. (C) Top 10 genes in each of the up‐ and down‐regulated DEGs. (D) Number distribution of independent sgRNAs in up‐regulated DEGs. (E) KEGG pathway enrichment analysis for up‐regulated DEGs. (F) GO functional enrichment analysis for up‐regulated DEGs.

To comprehensively investigate the drivers mediating abiraterone resistance, the hGeCKO library was used to screen for regulators related to abiraterone‐induced PCa resistance after knockout of 19,050 genes and 1864 miRNAs in 22Rv1 cells. Figure 1A displayed a schematic of the abiraterone‐treated cells used for high‐throughput sequencing analysis. The highly enriched sgRNAs in surviving cells represented genes targeted by these sgRNAs (down‐regulated DEGs) were associated with drug sensitivity compared to the initial cells (without abiraterone treatment), and vice versa. To obtain genes associated with abiraterone resistance, we selected 969 up‐regulated DEGs for analysis (Figure 1B). Figure 1C displayed the top 10 up‐ or down‐regulated DEGs. In addition, genes with independent sgRNA number ≥ 4 in the top 30 were further screened out (TBC1D3, USP17L11, ANXA8, TSPY1 and SPDYE5) (Figure 1D). Nine hundred and sixty nine up‐regulated DEGs were analysed for KEGG pathway and GO functional enrichment. KEGG revealed that these DEGs were mainly involved in the regulation of fatty acid metabolic pathways (such as fatty acid biosynthesis and degradation) (Figure 1E). GO revealed that these DEGs exerted a significant role in cellular immunoregulation (such as alpha‐beta T cell proliferation and MHC class II receptor activity) (Figure 1F). These results illustrate the reliability of hGeCKO library screening for abiraterone‐resistant genes.

### 3.2 Bioinformatics Identifies PPP1CA as a Key Abiraterone‐Resistant Gene

**FIGURE 2:**
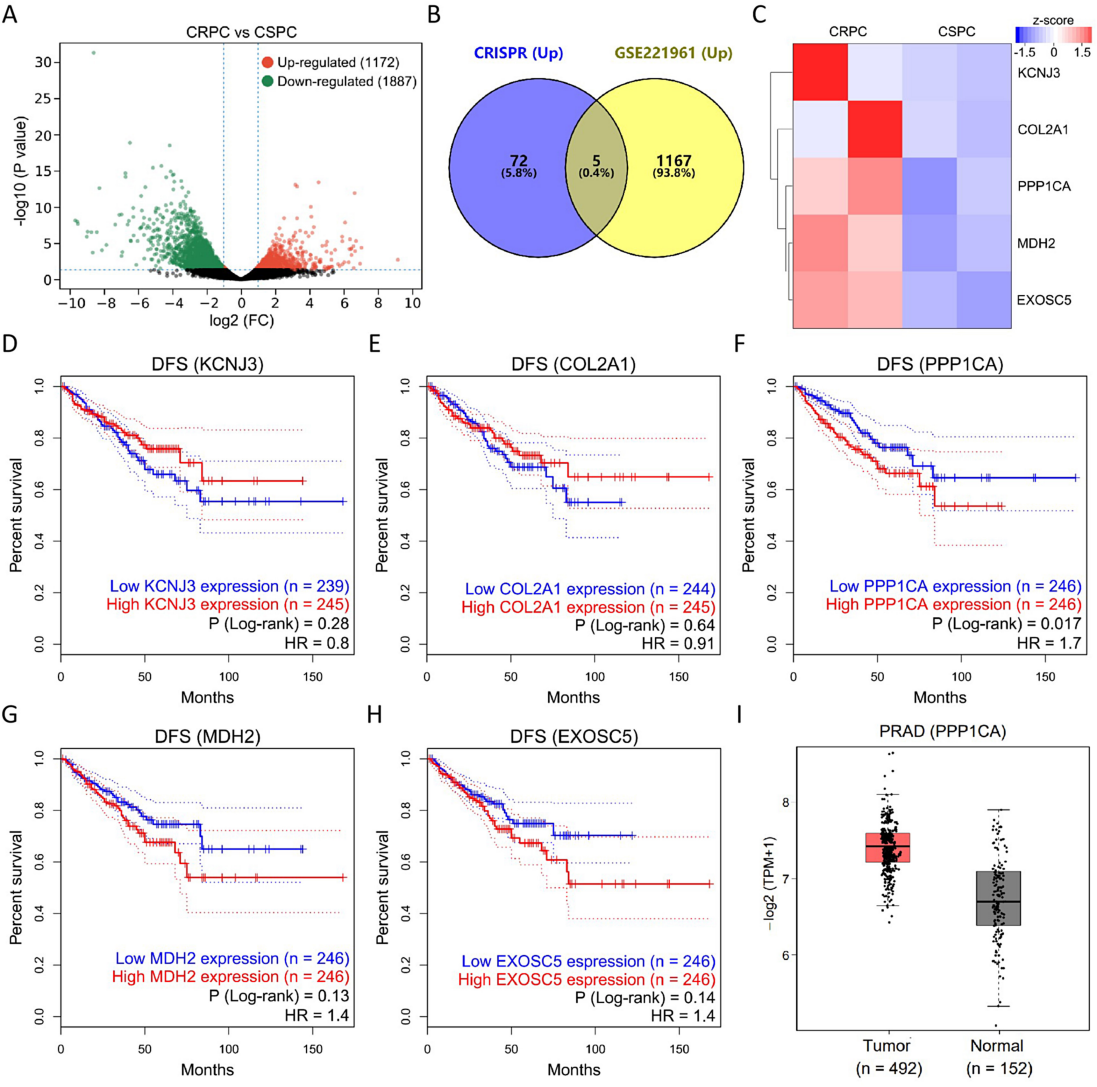
Identification for key resistance genes by bioinformatics. (A) Volcano diagram illustrating DEGs between CRPC and CSPC patients in the GSE221961 dataset. (B) Venn diagram illustrating the interaction of up‐regulated DEGs (*n* = 77) in CRISPR/Cas screening with up‐regulated DEGs (*n* = 1172) in CRPC patients. (C) Heat map illustrating the expression of five key resistance genes between CRPC and CSPC patients. (D) DFS of PCa patients with low (*n* = 239) and high (*n* = 245) KCNJ3 expression (*p* = 0.28, HR = 0.8). (E) DFS of PCa patients with low (*n* = 244) and high (*n* = 245) COL2A1 expression (*p* = 0.64, HR = 0.91). (F) DFS of PCa patients with low (*n* = 246) and high (*n* = 246) PPP1CA expression (*p* = 0.017, HR = 1.7). (G) DFS of PCa patients with low (*n* = 246) and high (*n* = 246) MDH2 expression (*p* = 0.13, HR = 1.4). (H) DFS of PCa patients with low (*n* = 246) and high (*n* = 246) EXOSC5 expression (*p* = 0.14, HR = 1.4). (I) Expression of PPP1CA in normal (*n* = 152) and PCa (*n* = 492) tissues from the PRAD cohort.

Clinically, PCa patients receiving ADT will inevitably develop CRPC, which means that the tumour can grow normally at an extremely low androgen level. At this point, the tumour is insensitive to ADT and is highly resistant to the drug. To identify the key genes driving PCa resistance, we explored the DEGs between CRPC and CSPC in the GSE221961 dataset, among which 1172 up‐regulated DEGs were associated with PCa resistance (Figure 2A). To further clarify the key abiraterone‐resistant genes, 77 up‐regulated DEGs (Table S2) enriched in KEGG and GO including TBC1D3, USP17L11, ANXA8, TSPY1 and SPDYE5 were interacted with 1172 up‐regulated DEGs in the GSE221961 dataset, and five key resistance genes (KCNJ3, COL2A1, PPP1CA, MDH2 and EXOSC5) were screened out (Figure 2B). Figure 2C displayed the expression of five key abiraterone‐resistant genes in CRPC and CSPC. Prognostic analysis found that high PPP1CA expression had a worse prognosis for DFS in PCa patients (HR = 1.7) (Figure 2D–H). Expression analysis also revealed the high PPP1CA expression in tumour tissues of PCa patients (Figure 2I). Thus, PPP1CA is a key gene driving abiraterone resistance in PCa.

### 3.3 PPP1CA Mediates Functional Regulation of Abiraterone‐Resistant 22Rv1 Cells

**FIGURE 3:**
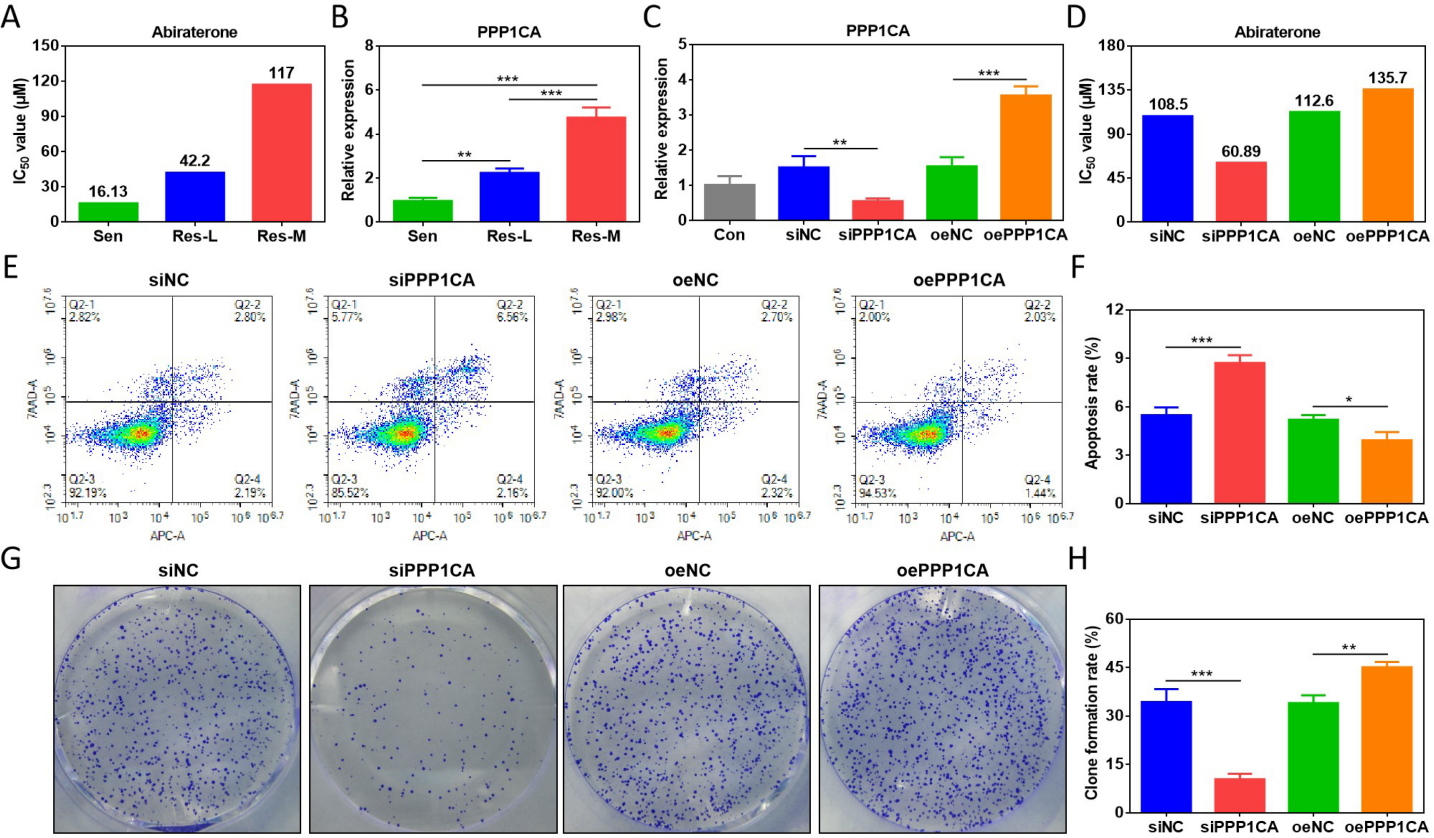
Effects of PPP1CA on abiraterone‐resistant 22Rv1 cell function. (A) IC_50_ of 22Rv1 cells with different resistance degrees. (B) Expression of PPP1CA in 22Rv1 cells with different resistance degrees. (C) Expression validation for PPP1CA silencing and overexpression in moderate‐resistant (Res‐M) cells. (D) Effects of PPP1CA silencing and overexpression on IC_50_ of Res‐M cells. (E) and (F) Effects of PPP1CA silencing and overexpression on apoptosis in Res‐M cells. (G) and (H) Effects of PPP1CA silencing and overexpression on clone formation in Res‐M cells (*p < 0.05, **p < 0.01, ***p < 0.001).

The role of PPP1CA in mediating abiraterone resistance was further explored. We induced the generation of abiraterone‐resistant cells by prolonged drug stimulation, culminating in low‐ (RI = 2.62) and moderate‐ (RI = 7.25) resistant 22Rv1 cells respectively (Figure 3A). Interestingly, PPP1CA expression was elevated with increasing degree of drug resistance (Figure 3B), suggesting a pro‐resistance role of PPP1CA. Then, cellular function was validated by gene silencing and overexpression. qRT‐PCR results demonstrated that siRNA and overexpression plasmid had a significant effect on PPP1CA expression in moderate‐resistant (Res‐M) cells (Figure 3C). Silencing PPP1CA inhibited cellular resistance to abiraterone, and overexpressing PPP1CA had the opposite effect (Figure 3D). Moreover, silencing PPP1CA significantly promoted cell apoptosis and inhibited clone formation, the opposite result was observed in overexpressing PPP1CA (Figure 3E–H). These results suggest that PPP1CA plays a key regulatory role in mediating abiraterone resistance, and targeting PPP1CA is an effective strategy against abiraterone resistance.

### 3.4 Nodularin‐R Inhibits PPP1CA, Potentially Leading to Anti‐CRPC Effects

**FIGURE 4:**
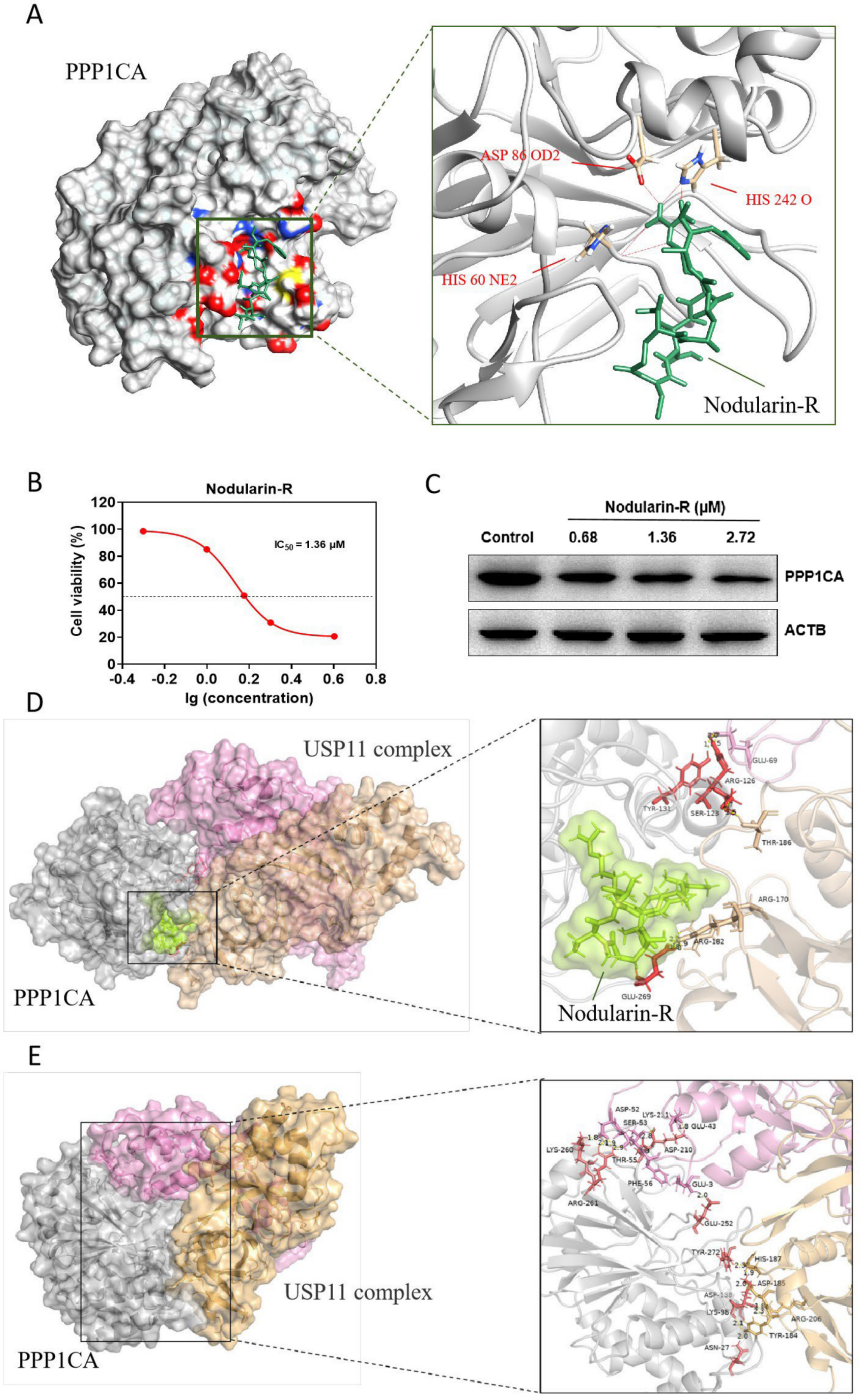
(A) Molecular docking result for nodularin‐R with PPP1CA. (B) IC_50_ of nodularin‐R on Res‐M cells. (C) Effects of different doses of nodularin‐R on PPP1CA expression in Res‐M cells. Molecular dynamics simulations of USP11‐PPP1CA‐Nodularin‐R complex (D) and USP11‐PPP1CA complex (E). Amino acids involved in hydrogen bonds from USP11 complex A (pink) and complex B (brown), as well as PPP1CA (grey), are shown in three‐letter code followed by their ordinal position in each chain. Nodularin‐R is represented by a green surface.

Nowadays, natural product extracts are proving to be an abundant source for small molecules (such as microcystins, nodularins and tautomycin) that effectively inhibit the catalytic activity of the serine/threonine protein phosphatase (PP) family, especially the PP1 and PP2A subtypes. Among them, nodularin‐R, a cyanotoxin produced by *Nodularia spumigena* with strong cytotoxicity, exerts the toxic effect by inhibiting PP1 and PP2A [20]. PPP1CA, as an important catalytic subunit of PP1, is largely inhibited by nodularin‐R. Further, potential targets for nodularin‐R were predicted by Swiss Target Prediction (STP) database (http://www.swisstargetprediction.ch/). We found that most of the targets were indeed focused on the PP family, including PPP1CA. In light of the absence of direct evidence regarding the interaction between nodularin‐R and PPP1CA, we opted to employ molecular docking to substantiate this potential interaction. Our findings revealed that nodularin‐R exhibited a capacity to bind to the PPP1CA protein through a concerted interplay of hydrophobic interactions with key residues such as TRP‐200, ASP‐214, GLN‐243, VAL‐244 and TYR‐266. Moreover, hydrogen bonds were identified between nodularin‐R and residues HIS‐60, ASP‐86, and HIS‐242, along with the presence of water bridges and a salt bridge (Figure 4A and Figure S2). Cell activity assays revealed that nodularin‐R was strongly cytotoxic to Res‐M cells (IC_50_ = 1.36 μM) (Figure 4B). WB results further demonstrated that nodularin‐R could inhibit PPP1CA protein in a dose‐dependent manner (Figure 4C). Given that USP11 has been reported to stabilise PPP1CA by deubiquitinating it and shielding it from proteasome‐mediated degradation [21], our aim was to deepen our understanding of how nodularin‐R influences the interaction between USP11 and PPP1CA. To achieve this, we employed molecular dynamics simulations using Gromacs2022.3 software. Following a 100 ns simulation, it was observed that the presence of nodularin‐R significantly influenced protein–protein interactions (PPI), resulting in a reduction in the number of hydrogen bonds from 10 to 5 (Figure 4D,E, Tables S3 and S4). Additionally, the total van der Waals force of the main chains within the USP11‐PPP1CA‐Nodularin‐R complex increased from −445.634 kcal/mol (in the USP11‐PPP1CA complex) to −396.537 kcal/mol. Furthermore, the free binding energy within the USP11‐PPP1CA‐Nodularin‐R complex increased from −68.92 ± 3.96 kcal/mol (in the USP11‐PPP1CA complex) to −28.13 ± 3.94 kcal/mol (Table S5). This observed phenomenon is likely attributed to the spatial obstruction induced by nodularin‐R (Figure 4D). The presence of nodularin‐R also has negative influence on internal/overall structural stability and the overall compactness of protein complex (Figure S3). These findings strongly indicate nodularin‐R's capability to inhibit PPP1CA protein, potentially leading to anti‐CRPC effects. Moreover, the diminished interaction between PPP1CA and USP11, along with the increased proteasome‐mediated degradation of PPP1CA induced by nodularin‐R, likely contribute significantly to this inhibition.

### 3.5 Synergistic Inhibitory Effects of Nodularin‐R on Abiraterone in Resistant 22Rv1 Cells

**FIGURE 5:**
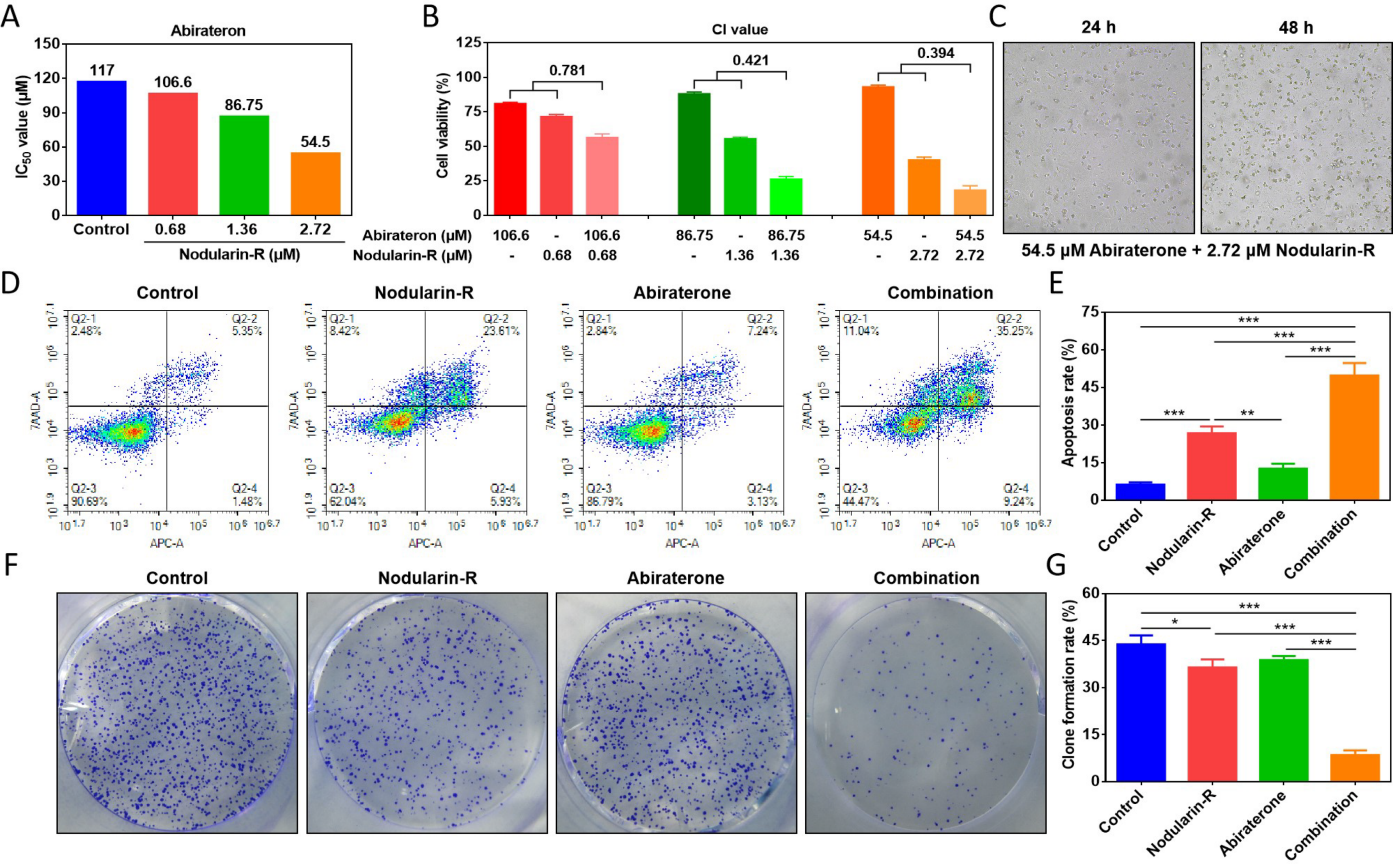
Anti‐tumour effects of nodularin‐R in combination with abiraterone in vitro. (A) IC_50_ of abiraterone on Res‐M cells after nodularin‐R pre‐treatment. (B) and (C) Effects of the combination for nodularin‐R and abiraterone on Res‐M cell viability. (D) and (E) Effects of the combination for nodularin‐R and abiraterone on apoptosis in Res‐M cells. (F) and (G) Effects of the combination for nodularin‐R and abiraterone on clone formation in Res‐M cells (*p < 0.05, **p < 0.01, ***p < 0.001).

Since nodularin‐R exhibited a potent inhibitory effect on the resistant target PPP1CA, there is reasonable to assume that it is potential to reverse the cellular resistance to abiraterone. To verify our speculation, the IC_50_ of abiraterone on Res‐M cells was assayed after pre‐treatment with different doses of nodularin‐R for 24 h. The results were consistent with our speculation that nodularin‐R could dose‐dependently increase the sensitivity of Res‐M cells to abiraterone (Figure 5A). Moreover, whether the strong cytotoxicity of nodularin‐R enhances the anti‐CRPC effects of abiraterone raises our concern. The combination of nodularin‐R and abiraterone had synergistic inhibitory effects in Res‐M cells, with the best synergistic effect of 2.72 μM nodularin‐R and 54.5 μM abiraterone (CI = 0.394) (Figure 5B,C). Cell function experiments also demonstrated that the combination of 2.72 μM nodularin‐R and 54.5 μM abiraterone significantly promoted apoptosis and inhibited clone formation (Figure 5D–G). Taken together, nodularin‐R can reverse the abiraterone resistance and enhance the inhibitory effects of abiraterone on Res‐M cells.

### 3.6 Synergistic Anti‐Tumour Effects of Nodularin‐R on Abiraterone In Vivo

**FIGURE 6:**
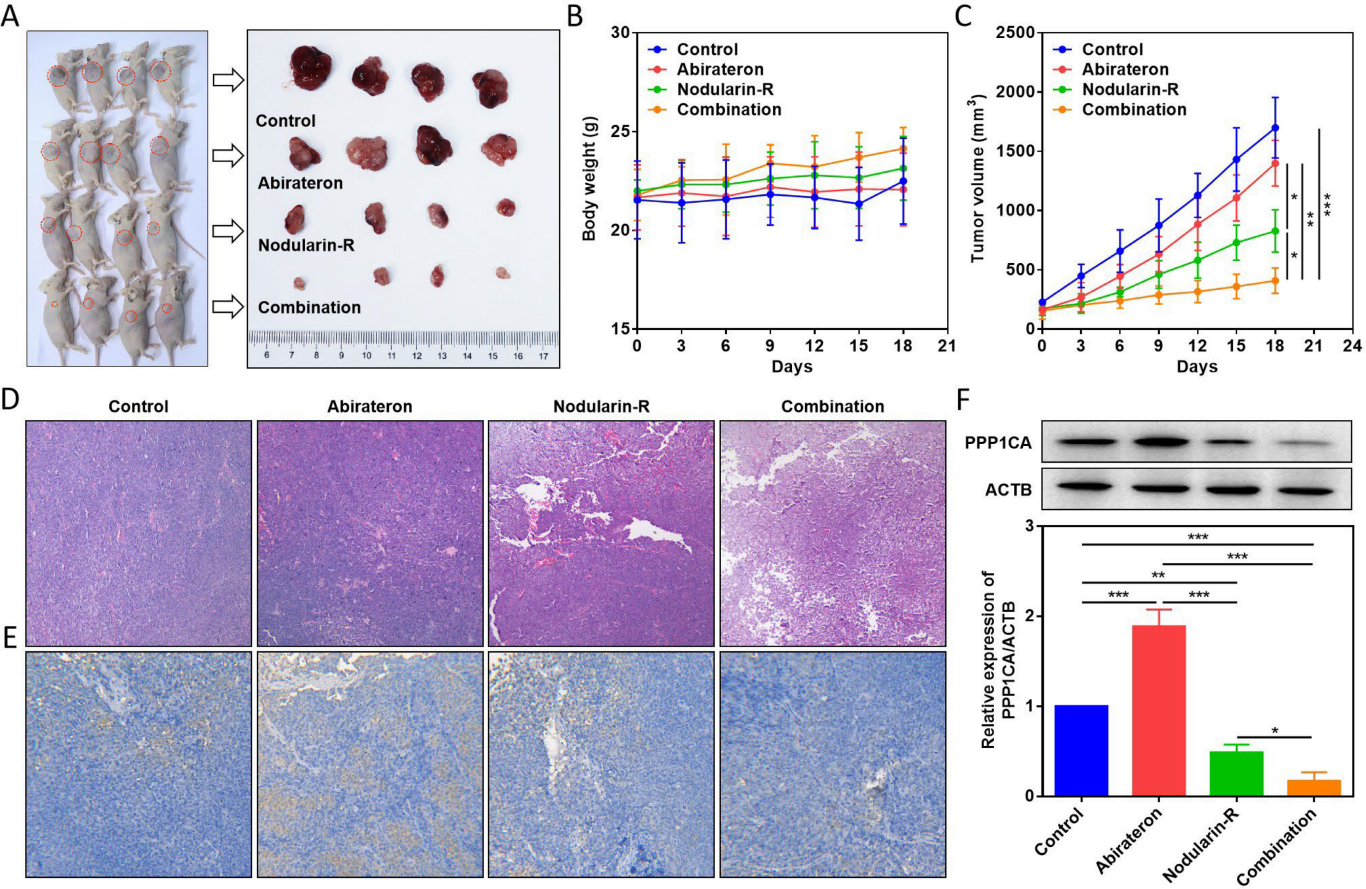
Anti‐tumour effects of nodularin‐R in combination with abiraterone in vivo. (A) Overall observation of tumour size in nude mice. (B) Monitoring of body weight changes in nude mice. (C) Monitoring of tumour volume changes in nude mice. (D) Observation (100 ×) of tumour histopathological changes by HE staining. (E) Observation (200 ×) of PPP1CA protein changes in tumour tissues by IHC staining. (F) WB assay for PPP1CA protein expression in tumour tissues (*p < 0.05, **p < 0.01, ***p < 0.001).

To better validate the synergistic anti‐tumour effects of nodularin‐R on abiraterone, we used xenografts to construct a subcutaneous tumour model in nude mice to observe the combined anti‐tumour effects of nodularin‐R and abiraterone. Clinically, CRPC patients are treated with 1000 mg abiraterone by oral administration daily. Based on the clinical dose of abiraterone, we performed a mouse/human surface area conversion method (conversion coefficient = 9.1) to determine the administered dose in nude mice at 150 mg/kg. Based on the dose ratio (20:1) of abiraterone to nodularin‐R in Res‐M cells, the administered dose of nodularin‐R in nude mice was determined at 7.5 mg/kg. To ensure an effective dose of nodularin‐R and avoid its toxicity to normal cells, we chose the intratumoural injection for therapy. We found that abiraterone or nodularin‐R therapy had no effects on body weight of nude mice (Figure 6B). Compared with abiraterone, nodularin‐R significantly inhibited tumour growth, and the inhibitory effects were most pronounced with the combination therapy (Figure 6A,C). HE staining revealed consistent results as well, in which the combination group exhibited significantly reduced number of tumour cells, enlarged cell gaps and increased cellular vacuole‐like necrosis (Figure 6D). IHC staining and WB results further revealed that abiraterone induced the PPP1CA expression in resistant tumour cells, which was significantly inhibited by nodularin‐R, with the most pronounced inhibitory effects in combination application (Figure 6E,F). These results demonstrate that PPP1CA mediates abiraterone resistance and nodularin‐R synergistically enhances the therapeutic effects of abiraterone by inhibiting PPP1CA.

## 4 Discussion

Clinically, adaptive changes of the androgen axis in CRPC patients receiving ADT lead to the emergence of acquired resistance. For example, incomplete blockade of androgen synthase P450c17 coupled with AKR1C3 overexpression leads to the emergence of intratumoral androgen synthesis [22, 23]. Thus, it is urgent to further understand the key mechanisms that mediate ADT drug resistance.

Screening of 22Rv1 cells for abiraterone resistance by CRISPR/Cas9 system revealed 969 up‐regulated DEGs enriched in surviving cells, indicating that these genes were highly related to abiraterone resistance, among which GLB1L2, COL27A1, SPDYE2, LGALS9B, PRUNE2, MAEA, ATP5G3, TBC1D3, USP17L11 and GADD45B ranked in the top 10. In addition, genes with independent sgRNA number ≥ 4 in the top 30 were further identified, including TBC1D3, USP17L11, ANXA8, TSPY1 and SPDYE5. COL27A1, PRUNE2, MAEA, TBC1D3, GADD45B, ANXA8 and TSPY1 have been confirmed to play a pro‐resistant role in various cancers [24, 25, 26, 27, 28, 29, 30], which reflects the reliability of the hGeCKO library screening to some extent. Further KEGG and GO enrichment revealed 77 genes enriched in various biosignals, especially in fatty acid metabolism and immunoregulation. Increasing studies have demonstrated that aberrant lipid metabolism drives CRPC formation [31]. For example, palmitate levels are higher in metastatic PCa patients who respond poorly to abiraterone, and Cav‐1 promotes hormone resistance by up‐regulating ACC1‐FASN and palmitate synthesis under ADT [32]. Partial resistance genes are also involved in the suppressive regulation of the tumour immune microenvironment. For example, GADD45B significantly correlates with and may stimulate T‐cell exhaustion in lung squamous cell carcinoma [33].

To further identify key resistance genes, 1172 up‐regulated DEGs in CRPC screened in the GSE221961 dataset were interacted with 77 DEGs enriched in KEGG and GO. We successfully screened five key genes, in which high PPP1CA expression had a poor prognosis for DFS in PCa patients. Despite the limited number of clinical samples available, we examined prostate cancer resistance‐related datasets in the GEO database but found no further information comparing clinical CRPC and CSPC samples. Nevertheless, both in vivo and in vitro experiments highlighted the role of PPP1CA in mediating resistance to abiraterone, strongly supporting our screening results. Moreover, PPP1CA was highly expressed in tumour tissues. Although the effects of PPP1CA on the prognosis in PCa patients have not been reported, the risk of its high expression can be verified in other cancer patients. In breast cancer, PPP1CA is highly expressed in tumour tissues and associated with poorer overall survival [34]. In bladder cancer, urinary PPP1CA levels can be used to detect tumour recurrence [35]. In glioblastoma, PPP1CA increases with increasing tumour grade and associated with poorer prognosis [36]. These studies suggest that PPP1CA may contribute to the PCa malignant phenotype. Consistent with our speculation that PPP1CA expression was higher in 22Rv1 cells with a higher degree of abiraterone resistance, and silencing PPP1CA significantly increased the sensitivity of Res‐M cells to abiraterone. In hepatocellular carcinoma, miR‐449a‐5p enhances the anti‐tumour effects of sorafenib by down‐regulating PPP1CA, including inhibiting cell proliferation and angiogenesis and inducing apoptosis [37]. In maxillary sinus squamous cell carcinoma, PPP1CA is directly regulated by miR‐874, and silencing PPP1CA inhibits cell proliferation and invasion [38]. Additionally, silencing PPPCCA significantly promoted apoptosis and inhibited clone formation in Res‐M cells, while overexpression had the opposite effects. These results suggest that PPP1CA promotes PCa progression and targeted inhibition of PPP1CA is a potential strategy to ameliorate abiraterone resistance.

Since there are commercially unavailable PPP1CA‐targeted inhibitors, we proposed to identify active monomers from natural products with potential inhibitory effects. Given the inhibition of protein phosphatase PP1/PP2A is structurally related to phycotoxins [39], the mode of interaction between nodularin‐R and PPP1CA was successfully predicted by molecular docking. Nodularin‐R is a phycotoxin with strong cytotoxicity, especially hepatotoxicity. Just 1–500 nM of nodularin‐R can differentially inhibit the viability of human hepatocellular carcinoma cells HepG2 [40]. Further validation revealed that nodularin‐R was also strongly toxic to Res‐M cells (IC_50_ = 1.36 μM) and dose‐dependently inhibited PPP1CA protein. In addition to the genotoxicity caused by oxidised purine‐induced oxidative DNA damage [41], the property of nodularin‐R in inducing cell apoptosis via PPP1CA inhibition provides the possibility for anti‐abiraterone resistance. We used molecular dynamics simulations to explore how nodularin‐R induces anti‐CRPC effects by affecting the interaction between USP11 and PPP1CA. Results showed that nodularin‐R reduced hydrogen bonds and increases van der Waals forces in the complex. This suggests nodularin‐R obstructs PPI, destabilising the complex. Additionally, nodularin‐R negatively impacts structural stability and compactness. These effects likely contribute to PPP1CA inhibition and anti‐CRPC effects by increasing proteasome‐mediated degradation and reducing interaction with USP11.

This was corroborated by the fact that nodularin‐R pre‐treatment reduced the IC_50_ of abiraterone on Res‐M cells. Further studies found that the combination of nodularin‐R and abiraterone exerted a synergistic inhibitory effect (CI < 1), with the combination of 2.72 μM nodularin‐R and 54.5 μM abiraterone being the most effective (CI = 0.394). The combination also demonstrated potent pro‐apoptotic and anti‐clone formation effects. Studies have revealed that cellular uptake of nodularin‐R is primarily via the organic anion transporters OATP1B1 and OATP1B3, which also mediate the transport of endogenous metabolites, hormones and drugs [42]. OATP1B3 (encoded by SLCO1B3) is highly expressed in some aggressive tumours and confers apoptosis resistance [43, 44], acting as a key regulator in mediating drug resistance. In PCa cells, OATP1B3 transports androgens intracellularly to maintain normal physiological properties. Abiraterone treatment induces SLCO1B3 expression driving ADT resistance in 22Rv1 cells and the 22Rv1 xenograft model [45]. Abiraterone‐induced SLCO1B3 up‐regulation provides a prerequisite for greater uptake of nodularin‐R by 22Rv1 cells, which may contribute to the synergistic anti‐tumour effects of both drugs. In addition, inhibition of AR‐independent signalling pathways (like Akt/mTOR pathway) by nodularin‐R may enhance the combined anti‐tumour effects [40]. These studies support the feasibility of nodularin‐R to ameliorate resistance and synergise potentiation on abiraterone. In vivo experiments revealed that PPP1CA expression was induced by abiraterone in resistant tumours, suggesting that PPP1CA is a key factor driving PCa resistance. In addition, the combination of nodularin‐R and abiraterone significantly inhibited tumour growth, further clarifying their synergistic anti‐CRPC effects. Overall, our study provides a novel molecular target and therapeutic strategy for CRPC therapy, especially for addressing abiraterone resistance.

In conclusion, we reveals a novel regulator PPP1CA driving abiraterone resistance. The natural product nodularin‐R ameliorates abiraterone resistance by inhibiting PPP1CA. The combination of nodularin‐R and abiraterone exerts synergistic anti‐CRPC effects.

## Author Contributions

**Yiqiao Huang:** conceptualization (equal), investigation (equal), methodology (equal), writing – original draft (equal). **Yi Cen:** conceptualization (equal), investigation (equal), methodology (equal), writing – original draft (equal). **Hualing Wu:** conceptualization (equal), investigation (equal), methodology (equal), writing – original draft (equal). **Guohao Zeng:** investigation (lead). **Zhengming Su:** investigation (equal). **Zhiming Zhang:** investigation (equal). **Shourui Feng:** formal analysis (equal), methodology (equal), software (lead), visualization (lead). **Xianhan Jiang:** funding acquisition (equal), project administration (equal), supervision (equal). **Anyang Wei:** conceptualization (equal), funding acquisition (lead), project administration (lead), supervision (lead).

## Ethics Statement

The authors have nothing to report.

## Consent

The authors have nothing to report.

## Conflicts of Interest

The authors declare no conflicts of interest.

## Supporting information


**Figure S1.** Screening for optimal dose of abiraterone in 22Rv1 cells. (A) Effects of different doses of abiraterone on cell viability. (B) OD statistics of the cells.
Figure S2. 2D nodularin‐R‐PPP1CA interaction diagram.
Figure S3. Nodularin‐R negatively impacts structural stability and compactness. (A) The gyrate values of USP11‐PPP1CA‐Nodularin‐R and USP11‐PPP1CA complexes. USP11‐PPP1CA complex (black) remained more stable than USP11‐PPP1CA‐nodularin‐R (red) throughout the simulation. (B) The SASA values of USP11‐PPP1CA‐Nodularin‐R and USP11‐PPP1CA during the simulation. Nodularin‐R has minimal impact on the surface features and stability of protein molecules. (C) The RMSD values of USP11‐PPP1CA‐Nodularin‐R and USP11‐PPP1CA complexes. The overall structure of USP11‐PPP1CA complex (black) remained more stable than USP11‐PPP1CA‐nodularin‐R (red) throughout the simulation. The RMSF values of (D) USP11 complex‐A, (E) USP11 complex‐B and (F) PPP1CA throughout the simulation. All protein molecules from USP11‐PPP1CA complex’s internal structural stability remained more stable than those from USP11‐PPP1CA‐Nodularin‐R.

Table S1.

## Data Availability

The data presented in this study are available on request from the corresponding authors.
