# Supplementary figures and images for: Nodularin‐R Synergistically Enhances Abiraterone Against Castrate‐ Resistant Prostate Cancer via PPP1CA Inhibition
Source: J Cell Mol Med. 2024 Nov 17;28(22):e70210. doi: 10.1111/jcmm.70210 (PMC11569623; doi:10.1111/jcmm.70210)

## Slide 1
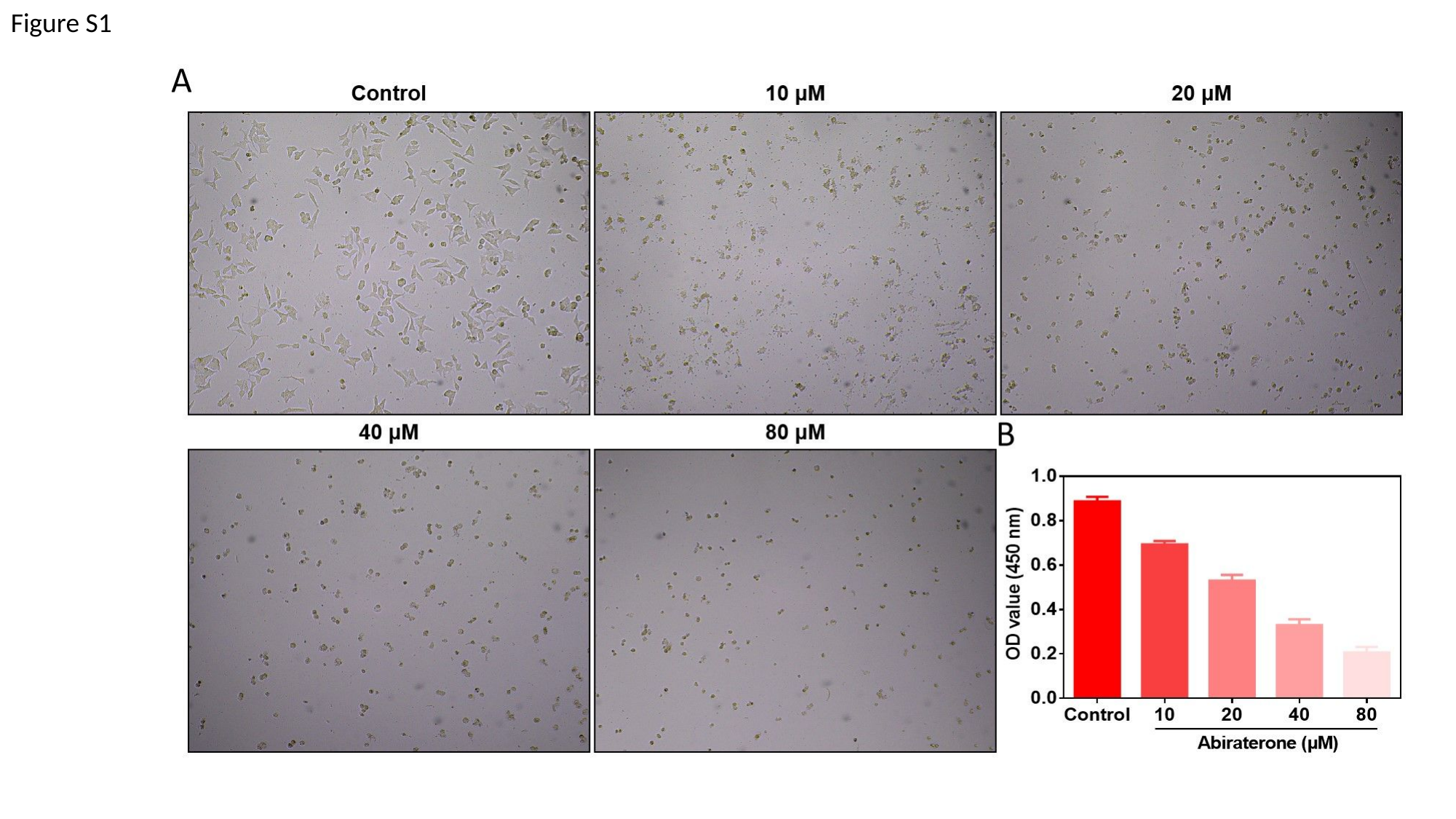

Figure S1

## Slide 2
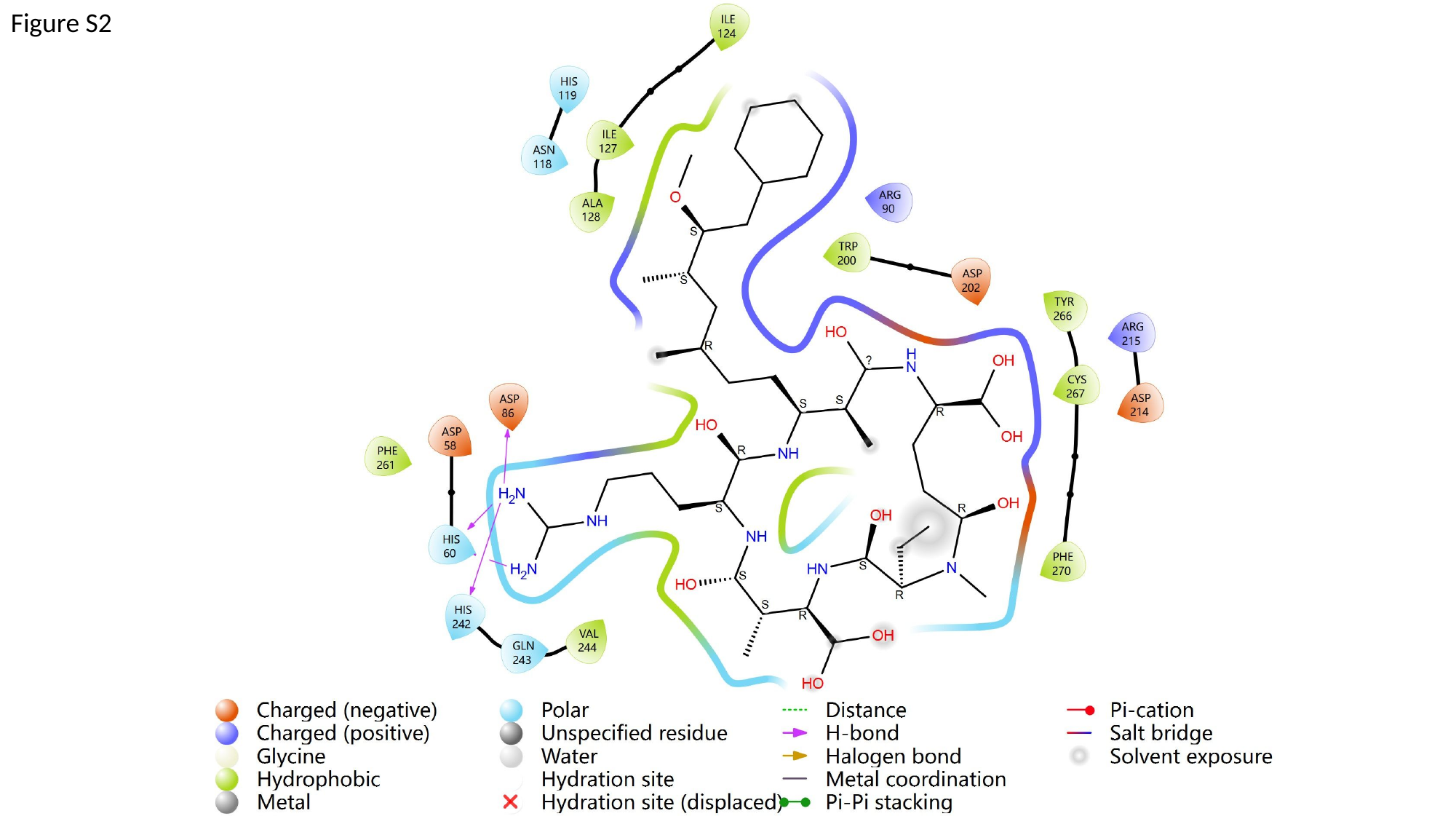

Figure S2

## Slide 3
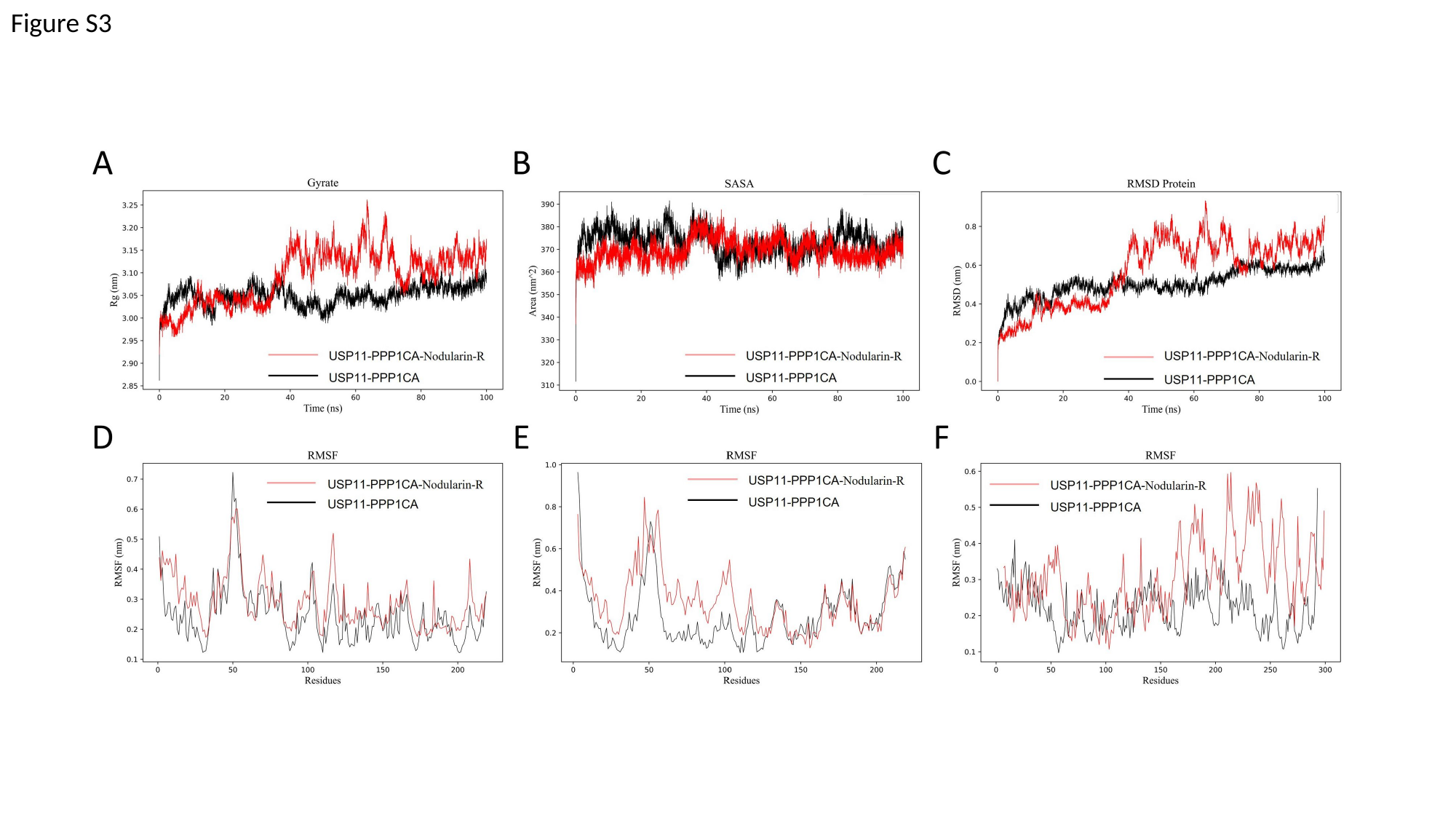

Figure S3

Supplement: Supplementary file 1 — Figure S1. Screening for optimal dose of abiraterone in 22Rv1 cells. (A) Effects of different doses of abiraterone on cell viability. (B) OD statistics of the cells. Figure S2. 2D nodularin‐R‐PPP1CA interaction diagram. Figure S3. Nodularin‐R negatively impacts structural stability and compactness. (A) The gyrate values of USP11‐PPP1CA‐Nodularin‐R and USP11‐PPP1CA complexes. USP11‐PPP1CA complex (black) remained more stable than USP11‐PPP1CA‐nodularin‐R (red) throughout the simulation. (B) The SASA values of USP11‐PPP1CA‐Nodularin‐R and USP11‐PPP1CA during the simulation. Nodularin‐R has minimal impact on the surface features and stability of protein molecules. (C) The RMSD values of USP11‐PPP1CA‐Nodularin‐R and USP11‐PPP1CA complexes. The overall structure of USP11‐PPP1CA complex (black) remained more stable than USP11‐PPP1CA‐nodularin‐R (red) throughout the simulation. The RMSF values of (D) USP11 complex‐A, (E) USP11 complex‐B and (F) PPP1CA throughout the simulation. All protein molecules from USP11‐PPP1CA complex’s internal structural stability remained more stable than those from USP11‐PPP1CA‐Nodularin‐R. [file JCMM-28-e70210-s001.pptx]
