# Supplementary material for: Nodularin‐R Synergistically Enhances Abiraterone Against Castrate‐ Resistant Prostate Cancer via PPP1CA Inhibition
Source: J Cell Mol Med. 2024 Nov 17;28(22):e70210. doi: 10.1111/jcmm.70210 (PMC11569623; doi:10.1111/jcmm.70210)
Supplement: Supplementary file 2 — Table S1. [file JCMM-28-e70210-s002.docx]

Table S1. Genome-wide CRISPR screening coverage

| Sample | Total sgRNA count | Sample sgRNA count | Sample / Total × 100 % |
| --- | --- | --- | --- |
| Control 1 | 123411 | 105932 | 85.84 % |
| Control 2 | 123411 | 106325 | 86.16 % |
| Control 3 | 123411 | 109594 | 88.80 % |
| Treatment 1 | 123411 | 25158 | 20.39 % |
| Treatment 2 | 123411 | 21649 | 17.54 % |
| Treatment 3 | 123411 | 21668 | 17.56 % |

Table S2. 77 up-regulated DEGs enriched in KEGG and GO

| No. | Genes | P value | Log2 (FC) | No. | Genes | P value | Log2 (FC) |
| --- | --- | --- | --- | --- | --- | --- | --- |
| 1 | CCL4L1 | 2.94E-02 | 7.02 | 40 | ACLY | 2.68E-02 | 1.09 |
| 2 | CD40 | 3.84E-02 | 9.24 | 41 | SDHC | 4.84E-02 | 9.18 |
| 3 | CFLAR | 4.94E-02 | 8.26 | 42 | MAL | 3.39E-02 | 9.14 |
| 4 | GADD45A | 3.58E-02 | 9.11 | 43 | NCMAP | 4.69E-03 | 3.39 |
| 5 | GADD45B | 2.47E-04 | 1.80 | 44 | PLP1 | 4.99E-02 | 8.69 |
| 6 | MAP3K14 | 4.40E-02 | 7.52 | 45 | KCND3 | 4.11E-02 | 2.01 |
| 7 | PLCG1 | 2.97E-02 | 8.76 | 46 | KCNJ3 | 4.59E-02 | 2.76 |
| 8 | TICAM2 | 4.52E-02 | 5.92 | 47 | KCNJ5 | 4.60E-02 | 9.19 |
| 9 | TRAF6 | 1.47E-02 | 4.60 | 48 | COL27A1 | 4.28E-05 | 9.68 |
| 10 | UBE2I | 3.01E-02 | 9.29 | 49 | COL2A1 | 7.34E-03 | 8.95 |
| 11 | ACADSB | 8.50E-03 | 8.85 | 50 | COL5A3 | 2.44E-02 | 4.17 |
| 12 | ACAT1 | 4.53E-02 | 9.20 | 51 | LUM | 4.26E-02 | 8.56 |
| 13 | ACSL3 | 4.48E-02 | 8.78 | 52 | MPP5 | 2.46E-02 | 6.62 |
| 14 | CBR4 | 1.81E-02 | 7.38 | 53 | PRKCI | 2.21E-02 | 2.31 |
| 15 | ECHS1 | 9.09E-03 | 9.18 | 54 | CTNNBL1 | 3.48E-02 | 9.26 |
| 16 | HSD17B8 | 1.32E-02 | 8.75 | 55 | ISY1 | 8.54E-03 | 3.68 |
| 17 | BMP2 | 1.72E-02 | 8.11 | 56 | U2AF2 | 1.24E-02 | 2.30 |
| 18 | WNT10A | 1.24E-02 | 9.49 | 57 | DIS3L | 3.26E-02 | 5.41 |
| 19 | WNT3 | 3.19E-02 | 9.28 | 58 | EXOSC5 | 6.59E-03 | 9.15 |
| 20 | WNT5B | 2.79E-02 | 9.31 | 59 | EXOSC7 | 1.24E-02 | 8.93 |
| 21 | WNT7A | 1.74E-02 | 9.42 | 60 | CD81 | 3.31E-02 | 8.14 |
| 22 | CD28 | 4.07E-02 | 8.92 | 61 | LGALS9 | 2.55E-02 | 8.63 |
| 23 | FAS | 6.19E-03 | 9.62 | 62 | LGALS9B | 1.54E-04 | 9.60 |
| 24 | HLA-DOA | 3.57E-02 | 7.21 | 63 | LGALS9C | 1.94E-02 | 9.74 |
| 25 | HLA-DPA1 | 3.83E-02 | 3.50 | 64 | XCL1 | 1.01E-02 | 4.17 |
| 26 | HLA-DQB1 | 4.46E-03 | 5.05 | 65 | NF1 | 1.81E-02 | 8.68 |
| 27 | DGAT1 | 1.74E-02 | 9.42 | 66 | PPP1CA | 3.27E-02 | 8.51 |
| 28 | PLA2G2F | 3.47E-02 | 6.44 | 67 | PPP2R1A | 4.29E-02 | 8.78 |
| 29 | SLC27A1 | 4.84E-02 | 8.45 | 68 | RET | 2.95E-02 | 4.65 |
| 30 | SLC27A4 | 1.13E-02 | 3.43 | 69 | APP | 7.10E-04 | 4.20 |
| 31 | ADH1B | 1.52E-02 | 4.04 | 70 | C1QA | 1.51E-02 | 4.20 |
| 32 | AKR1A1 | 4.93E-02 | 8.56 | 71 | C1QL1 | 1.90E-02 | 8.78 |
| 33 | HAGH | 3.19E-02 | 0.71 | 72 | FARP2 | 2.94E-02 | 8.54 |
| 34 | MDH1 | 1.44E-02 | 9.45 | 73 | TBC1D3 | 4.75E-06 | 3.69 |
| 35 | MDH2 | 3.29E-02 | 3.91 | 74 | USP17L11 | 3.43E-04 | 2.63 |
| 36 | CS | 3.41E-03 | 5.99 | 75 | ANXA8 | 3.84E-04 | 6.95 |
| 37 | ECI1 | 4.70E-02 | 4.68 | 76 | TSPY1 | 1.05E-03 | 0.81 |
| 38 | IL12B | 4.90E-02 | 6.04 | 77 | SPDYE5 | 1.19E-03 | 5.50 |
| 39 | EPX | 4.04E-02 | 9.18 |  |  |  |  |

Table S3. Hydrogen bonds between USP11 complex and PPP1CA with Nodularin-R interacted

| Hydrogen bonds | | | |
| --- | --- | --- | --- |
| No. | PPP1CA with Nodularin-R | Dist. [Å] | USP11 complex A |
| 1 | C:GLU 269 [OE1] | 1.90 | A:ARG 182 [HH12] |
| 2 | C:GLU 269 [OE2] | 1.80 | A:ARG 170 [HH22] |
| No. | PPP1CA with Nodularin-R | Dist. [Å] | USP11 complex B |
| 1 | C:ARG 126 [HH21] | 1.97 | B:GLU 69 [OE1] |
| 2 | C:TYR 131 [HH] | 1.89 | B:GLU 69 [OE2] |
| 3 | C:ARG 126 [HH12] | 2.12 | B:HIS 103 [O] |

Table S4. Hydrogen bonds between USP11 complex and PPP1CA

| Hydrogen bonds | | | |
| --- | --- | --- | --- |
| No. | PPP1CA | Dist. [Å] | USP11 complex A |
| 1 | C:ASN 27 [HD22] | 1.97 | A:TYR 184 [OH] |
| 2 | C:LYS 98 [HZ3] | 2.03 | A:ASP 185 [OD2] |
| 3 | C:LYS 98 [O] | 2.13 | A:TYR 184 [HH] |
| 4 | C:ASP 138 [OD2] | 1.81 | A:ARG 206 [HH12] |
| 5 | C:ASP 138 [OD2] | 2.30 | A:ARG 206 [HH22] |
| No. | PPP1CA | Dist. [Å] | USP11 complex B |
| 1 | C:LYS 211 [HZ3] | 1.80 | B:GLU 43 [OE2] |
| 2 | C:LYS 260 [HZ3] | 1.77 | B:ASP 52 [OD1] |
| 3 | C:ARG 261 [HH22] | 1.88 | B:ASP 52 [OD2] |
| 4 | C:GLU 252 [OE1] | 2.02 | B:GLU 3 [H2] |
| 5 | C:ASP 210 [O] | 1.92 | B:PHE 56 [H] |

Table S5. Comparison of MM/PBSA results for the USP11-PPP1CA-Nodularin-R and USP11-PPP1CA complexes

| Energy component | USP11-PPP1CA-Nodularin-R | USP11-PPP1CA |
| --- | --- | --- |
| ΔBOND | 0 ± 0 | 0 ± 0 |
| ΔANGLE | 0 ± 0 | 0 ± 0 |
| ΔDIHED | 0 ± 0 | 0 ± 0 |
| ΔVDWAALS | -118.71 ± 11.37 | -193.5 ± 2.91 |
| ΔEEL | 1260.59 ± 82.51 | 1868.49 ± 39.06 |
| Δ1-4 VDW | 0 ± 0 | 0 ± 0 |
| Δ1-4 EEL | 0 ± 0 | 0 ± 0 |
| ΔEGB | -1149.95 ± 72.02 | -1723.8 ± 36.37 |
| ΔESURF | -20.06 ± 1.01 | -20.11 ± 0.38 |
| ΔGGAS | 1141.88 ± 73.51 | 1674.99 ± 37.6 |
| ΔGSOLV | -1170.01 ± 72.58 | -1743.92 ± 36.53 |
| ΔTOTAL | -28.13 ± 3.94 | -68.92 ± 3.96 |

ΔVDWAALS: van der Waals energy

ΔEEL: electrostatic energy

ΔEGB: polar solvent solvation energy

ΔESURF: nonpolar solvent solvation energy

ΔGGAS: gas-phase molecular mechanics energy = ΔVDWAALS + ΔEEL

ΔGSOLV: solvation energy = ΔEGB + ΔESURF

ΔTOTAL: total energy = ΔGGAS + ΔGSOLV

All units are reported in kcal/mol. Using temperature = 298.15 K
